# Development and Validation of a Novel Microbiome-Based Biomarker of Post-antibiotic Dysbiosis and Subsequent Restoration

**DOI:** 10.3389/fmicb.2021.781275

**Published:** 2022-01-04

**Authors:** Ken Blount, Courtney Jones, Dana Walsh, Carlos Gonzalez, William D. Shannon

**Affiliations:** ^1^Rebiotix Inc., a Ferring Company, Roseville, MN, United States; ^2^BioRankings LLC, St. Louis, MO, United States

**Keywords:** intestinal microbiota, dysbiosis, antibiotics, biomarker, *Clostridioides difficile* infection

## Abstract

**Background:** The human gut microbiota are important to health and wellness, and disrupted microbiota homeostasis, or “dysbiosis,” can cause or contribute to many gastrointestinal disease states. Dysbiosis can be caused by many factors, most notably antibiotic treatment. To correct dysbiosis and restore healthier microbiota, several investigational microbiota-based live biotherapeutic products (LBPs) are in formal clinical development. To better guide and refine LBP development and to better understand and manage the risks of antibiotic administration, biomarkers that distinguish post-antibiotic dysbiosis from healthy microbiota are needed. Here we report the development of a prototype Microbiome Health Index for post-Antibiotic dysbiosis (MHI-A).

**Methods:** MHI-A was developed and validated using longitudinal gut microbiome data from participants in clinical trials of RBX2660 and RBX7455 – investigational LBPs in development for reducing recurrent *Clostridioides difficile* infections (rCDI). The MHI-A algorithm relates the relative abundances of microbiome taxonomic classes that changed the most after RBX2660 or RBX7455 treatment, that strongly correlated with clinical response, and that reflect biological mechanisms believed important to rCDI. The diagnostic utility of MHI-A was reinforced using publicly available microbiome data from healthy or antibiotic-treated populations.

**Results:** MHI-A has high accuracy to distinguish post-antibiotic dysbiosis from healthy microbiota. MHI-A values were consistent across multiple healthy populations and were significantly shifted by antibiotic treatments known to alter microbiota compositions, shifted less by microbiota-sparing antibiotics. Clinical response to RBX2660 and RBX7455 correlated with a shift of MHI-A from dysbiotic to healthy values.

**Conclusion:** MHI-A is a promising biomarker of post-antibiotic dysbiosis and subsequent restoration. MHI-A may be useful for rank-ordering the microbiota-disrupting effects of antibiotics and as a pharmacodynamic measure of microbiota restoration.

## Introduction

Disruptions to the human gut microbiota, known as dysbiosis, can contribute to gastrointestinal, neurologic, epithelial, genitourinary, and oncological disorders (Duvallet et al., 2017). To counteract and correct dysbiosis, several microbiota-based live biotherapeutic products (LBPs) are now in formal clinical development. Accordingly, biomarkers to identify and monitor treatment of dysbiosis are a health care priority. At present, measurements of dysbiosis are multivariate, complex, and often narrowly defined to a single study population. There may also be multiple dysbiosis patterns, with each related to a specific cause(s) or health care risk(s).

The most commonly cited cause of dysbiosis is antibiotic treatment, with clearly associated healthcare risks like *Clostridioides difficile* infections (CDI) or infections by multidrug-resistant *Enterobacteriaceae* or *Enterococci* (Brown et al., 2013). Post-antibiotic dysbiosis was first characterized *via* culturing methods (Edlund et al., 1997), followed by quantitative PCR (Tannock et al., 2010), followed by sequencing of the collective genomes of the entire microbiota community – known as the microbiome (Costea et al., 2018). Microbiome characterization provides the most complete picture of post-antibiotic dysbiosis, but the associated data are multivariate and compositional and require specialized expertise and statistical tools uncommon to diagnostic settings (La Rosa et al., 2012). The aim of the present study was to develop a simple univariate microbiome-based biomarker of post-antibiotic dysbiosis and subsequent restoration that could support diagnostic decisions. To that end, we report a novel Microbiome Health Index for post-Antibiotic dysbiosis (MHI-A), developed and validated using data from controlled clinical trials of investigational LBPs in development for reducing rCDI recurrence. We also present validating data from multiple healthy or antibiotic-treated populations. The implications and potential utility of MHI-A will be discussed.

## Materials and Methods

### Microbiome Data Included in the Analysis

Microbiome composition data were included for fecal samples collected from participants in three clinical trials of investigational microbiota-based LBPs being developed to reduce rCDI recurrence. RBX2660 – an investigational LBP formulated as a liquid suspension – was evaluated in the PUNCH CD2 and PUNCH Open Label trials (Dubberke et al., 2018; Orenstein et al., 2019). RBX7455 – a non-frozen, lyophilized, orally administered investigational LBP – was evaluated in a phase 1 trial (Khanna et al., 2020). All three trials enrolled participants with a documented history of rCDI, and all three had a prespecified clinical success endpoint of absence of rCDI recurrence at 8 weeks after the last received treatment. All included the analysis of microbiome changes after investigational treatment as an exploratory endpoint, the results of which are reported elsewhere (Khanna et al., 2016b; Blount et al., 2019; Orenstein et al., 2019). Participants were asked to provide stool samples prior to and at time points after treatment, and the samples were sequenced using 16S for PUNCH CD2, shallow shotgun for PUNCH Open-Label, and whole genome shotgun for the RBX7455 phase 1. Operational taxonomic units (OTU) data were calculated from sequence data using standard methods and a proprietary pipeline and database (Diversigen, MN, United States), and relative taxonomic abundances were calculated from OTU.

Human Microbiome Project data, sequenced using 16S, were accessed from the HMP data portal (https://portal.hmpdacc.org; Human Microbiome Project Consortium, 2012). Data from a Scandinavian healthy adult cohort (PopCol) were accessed from the European Nucleotide Archive (ENA; Hugerth et al., 2020). Sequencing data from fecal microbiota transplant (FMT) donors were accessed from three published studies *via* supplementary information (Weingarden et al., 2015), the European Bioinformatics Institute (Khanna et al., 2017), or the National Center for Biotechnology Information, NCBI (Staley et al., 2018). Sequencing data for antibiotic-treated individuals were from three controlled clinical studies, sourced from NCBI (Zaura et al., 2015; Thorpe et al., 2018), or ENA (Palleja et al., 2018). To minimize interstudy variability, all accessed sequencing data were processed to OTU using the same analysis pipeline and database (Diversigen, Minneapolis, MN) as was used for RBX2660 and RBX7455 trials, except the HMP set which was only accessible as OTU data.

### Statistical Analyses and MHI Derivation

Group relative abundances (π) with confidence limits were generated by fitting a Dirichlet-multinomial (DM) distribution to OTU data using maximum likelihood estimation (La Rosa et al., 2012). Dirichlet-multinomial Recursive Partitioning (DM-RPart; Yang et al., 2019) was used to regress taxa count data onto time of sample to identify how the microbiome changed from baseline to post-treatment follow-up. Samples within a terminal node of the DM-RPart tree identify homogeneous subgroups and indicate which taxa are different across the terminal nodes. We display these differences by heat charts of the taxa compositions. This DM-RPart analysis identified 4 taxa which separated microbiome compositions into baseline samples representing dysbiosis and later post-treatment or RBX2660 samples representing a return toward a normal microbiome. Bacilli and Gammaproteobacteria were dominant classes at baseline (dysbiosis), and Bacteroidia and Clostridia were dominant classes post-treatment.

Univariate logistic regression was used to test each class separately to estimate the probability a sample is dysbiotic. Regression coefficients, nominal *p* values, and odds ratios showed that each class by itself separated the baseline and RBX2660 groups. The odds ratios for Gammaproteobacteria and Bacilli indicated high value of these classes to predict post-antibiotic dysbiosis and low odds ratios for Bacteroidia and Clostridia indicated high value of these classes to predict healthy or non-dysbiotic samples (Supplementary Table S1). Several multivariate logistic regression models were fit to these taxa with the ratio of relative abundances Gammaproteobacteria + Bacilli divided by the relative abundances of Bacteroidia + Clostridia being the best fit (Supplementary Table S2). The inverse of this was used as the MHI-A ratio as a predictor of non-dysbiotic.

Receiver operating characteristic (ROC) analyses were conducted within GraphPad Prism using baseline and RBX2660 MHI-A data from the PUNCH CD2 trial. All pairwise hypothesis testing between MHI-A data groups, which used Mann–Whitney tests unless otherwise specified, was conducted within GraphPad Prism.

## Results

### Development of MHI-A

The MHI-A was designed to differentiate post-antibiotic dysbiosis from healthy microbiota. Literature teaches that many antibiotics reduce the relative abundance of Bacteroidetes and Firmicutes phyla compared to healthy populations, with a concomitant increase in Proteobacteria (reviewed in Lange et al., 2016). At the class level Clostridia and Bacteroidia are reduced the most, whereas Bacilli, also Firmicutes phylum, are often increased. The Gammaproteobacteria class is often increased the most, within which Enterobacterales and Pseudomonadales families increase most.

**Figure 1:**
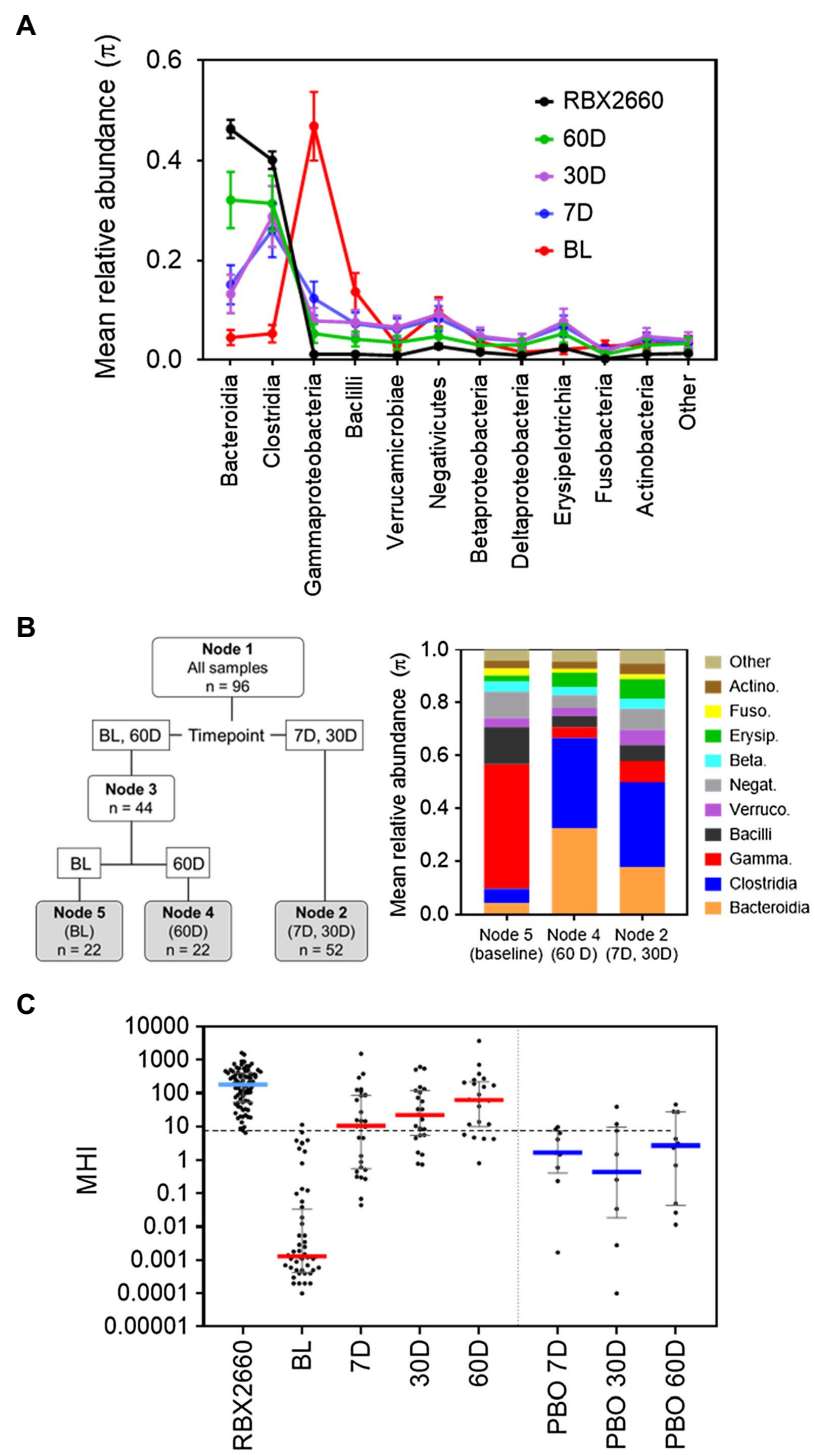
Development of MHI-A. **(A)** Mean relative abundance (π) at the class level for samples from the PUNCH CD2 trial, denoted as before treatment (BL), 7, 30, and 60 days (7 D, 30 D, and 60 D) after treatment, or the administered doses of RBX2660 investigational product. Means (π) with upper and lower confidence intervals were calculated based on maximum likelihood estimate fit to a Dirichlet multinomial distribution, with classes comprising less than 3% relative abundance at all time points combined as “Other.” **(B)** Dirichlet-multinomial Recursive Partitioning (DM-RPart) was fit to regress the *N* = 96 microbiome composition data from the PUNCH CD2 trial onto BL, 7 D, 30 D, and 60-day timepoints. Ten-fold cross-validation was used to fit the optimal tree which resulted in 3 terminal nodes: BL (*n* = 22 samples), 7 D and 30 D (*n* = 52 samples), and 60 D (*n* = 22 samples). The taxa abundances for the three terminal nodes are shown in the color bar chart. This indicates that at BL Bacilli and Gammaproteobacteria were the top two dominant classes, and in post-treatment timepoints Bacteroidia and Clostridia became the dominant classes. This suggests these taxa can be used to distinguish representative post-antibiotic dysbiosis (BL) from healthy (RBX2660) populations. **(C)** MHI-A values for PUNCH CD2 samples, shown on a logarithmic scale as median and interquartile ranges with individual samples overlaid. Timepoints shown are baseline (BL), 7 days (7 D), 30 days (30 D), and 60 days (60 D) after treatment with RBX2660 or placebo (PBO). MHI-A values for the administered doses of RBX2660 investigational product are also shown (RBX2660). The dotted line shows the MHI-A = 7.2 threshold, above which MHI-A values correspond to a healthy based on ROC analysis.

We observed similar differences between antibiotic-treated and healthy among participants in the PUNCH CD2 controlled clinical trial of the investigational LBP RBX2660 (NCT02299570; Dubberke et al., 2018; Blount et al., 2019). In PUNCH CD2, patients with a recent episode of recurrent CDI (rCDI) were administered RBX2660 after a standard-of-care antibiotic (metronidazole, fidaxomicin, or vancomycin), with the clinical aim of reducing rCDI recurrence. For developing MHI-A, participant fecal samples collected prior to RBX2660 administration (baseline) were used as a representative post-antibiotic population because they had just completed antibiotic treatment. RBX2660 doses administered to patients were used as a representative healthy population, since they were manufactured from stool donations from healthy donors who had not recently received antibiotics. At baseline, Bacteroidia- and Clostridia-class bacteria were decreased, whereas Gammaproteobacteria and Bacilli were increased compared to RBX2660 (Figure 1A). The baseline compositions were similar to other post-CDI-antibiotic populations (Tannock et al., 2010; Louie et al., 2012; Palleja et al., 2018), and the RBX2660 compositions were similar to other healthy cohorts (Human Microbiome Project Consortium, 2012).

A recursive partitioning analysis confirmed that baseline and RBX2660 compositions were statistically distinguishable by their relative abundances of the Bacteroidia, Clostridia, Gammaproteobacteria, and Bacilli classes (Figure 1B), and these four classes had the highest relative abundances among the combined population. Of note, these classes also have biological relevance to post-antibiotic dysbiosis versus healthy, in that Bacteroidia and Clostridia are attributed with beneficial functions known to be disrupted by antibiotics – colonization resistance, immune modulation, bile acid, and short chain fatty acid metabolism, and Gammaproteobacteria and Bacilli can proliferate and cause infection after antibiotics (Pickard et al., 2017; Ducarmon et al., 2019). Logistic regression analysis indicated that the log relative abundances of Bacteroidia and Clostridia positively correlated with healthy, whereas Gammaproteobacteria and Bacilli negatively correlated. This relationship was algebraically simplified to the following ratio:


$$MHI-A=\frac{\left[\begin{array}{l} RA,\\ Bacteroidia \end{array}\right]+\left[\begin{array}{l} RA,\\ Clostridia \end{array}\right]}{\left[\begin{array}{l} RA,\\ Gammaproteobacteria \end{array}\right]+\left[\begin{array}{l} RA,\\ Bacilli \end{array}\right]}$$

Where *RA* indicates relative abundance. Among baseline samples (representative of post-antibiotic dysbiosis), the median MHI-A was 0.0013, whereas the median MHI-A for RBX2660 was 183 (Figure 1C; Supplementary Table S3). To evaluate the diagnostic utility of MHI-A as a binary classifier of healthy versus post-antibiotic dysbiosis, receiver operating characteristic (ROC) analysis was conducted, which compares sensitivity versus specificity across a range of classifier cut points. The cut point of MHI-A = 7.2 was determined as optimal, because it had the highest sensitivity (98%) and the highest specificity (98%; Supplementary Table S4; Supplementary Figure S1), and the area under the ROC curve (AUROC) was 0.99, indicating a high diagnostic accuracy.

### MHI-A After RBX2660 Administration in PUNCH CD2

Voluntary stool samples from participants were also collected after treatment with RBX2660 at pre-determined timepoints. At 7, 30, and 60 days after treatment, the median MHI-A for RBX2660 treatment responders was significantly higher than baseline (*p* < 0.001), and the majority were > 7.2 (Figure 1B; Supplementary Table S3). Among the subset of 14 RBX2660 responders from whom all of baseline, 7-, 30-, and 60-day samples were received, MHI-A increased by an average of 87,000-fold from baseline to 7 days, 33-fold from seven to 30 days, and 4-fold from 30 to 60 days (Supplementary Figure S2). Responders in the placebo-treated arm also showed MHI-A restoration, but the majority remained lower than 7.2 at all timepoints, with a median MHI-A significantly lower than RBX2660 responders at each timepoint (*p* < 0.05). Analysis of non-responders (CDI recurrence) was limited because most recurred prior to the earliest 7-day time point, at which point participants received additional antibiotic and/or RBX2660 treatment and were therefore excluded from subsequent microbiome analysis. Among the five 7-day and two 30-day samples that were included from non-responders, most had MHI-A < 7.2 (Supplementary Table S3). Overall, MHI-A had high accuracy to distinguish antibiotic-treated from healthy and was effective marker of microbiome changes that correlated with RBX2660 treatment response in PUNCH CD2.

### MHI-A in Additional Investigational LBP Trials

**Figure 2:**
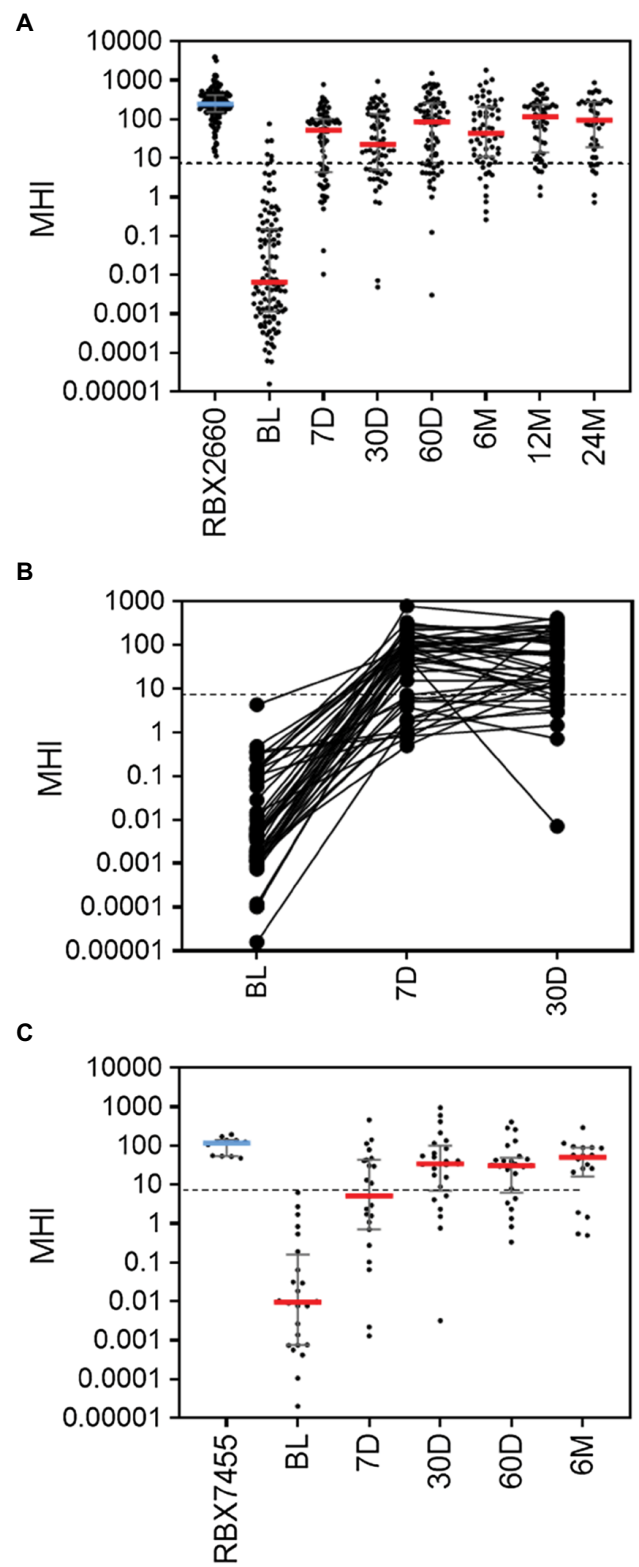
MHI-A values for participants in the PUNCH Open Label trial of RBX2660 and a Phase 1 trial of RBX7455 **(A)** MHI-A values for all PUNCH Open Label samples, shown as individual sample values with median and interquartile ranges. Timepoints shown are baseline (BL), 7 days (7 D), 30 days (30 D), 60 days (60 D), 6 months (6 M), 12 months (12 M), and 24 months (24 M) after treatment with RBX2660. MHI-A values for the administered doses of RBX2660 investigational product are also shown (RBX2660). The dotted line shows the MHI-A = 7.2 threshold, above which MHI-A values correspond to a healthy based on ROC analysis. **(B)** Longitudinal within-participant MHI-A values for the subset of RBX2660-treated responders from whom all three displayed timepoints were received. **(C)** MHI-A values for all RBX7455 responder samples received and for administered RBX7455 drug product.

To further validate MHI-A, data from two additional controlled trials were assessed, including the PUNCH Open Label trial of RBX2660 and a phase 1 trial of RBX7455 – a non-frozen, orally-administered investigational microbiota-based live biotherapeutic. In PUNCH Open-Label, 79% of participants who received RBX2660 were CDI recurrence-free at 8 weeks after treatment, and responders’ microbiome compositions changed significantly from before to after treatment, as determined using shallow shotgun sequencing (Orenstein et al., 2019). The median MHI-A values for participants at baseline and RBX2660 investigational product were comparable to the respective groups in PUNCH CD2 (Figure 2A; Supplementary Table S3). By 7 days after treatment, the median and majority of RBX2660 responders MHI-A shifted >7.2 and remained so to at least 24 months after treatment. Among the subset of 44 responders from whom all of baseline, 7-, and 30-day samples were received, MHI-A increased by an average of 65,000-fold from baseline to 7 days and 11-fold between seven and 30 days (Figure 2B). Analysis of non-responders was limited because the majority recurred prior to the earliest 7-day time point, and MHI-A only increased by an average of 820-fold among those which recurred later. Thus, PUNCH Open-Label MHI-A results were consistent with PUNCH CD2.

In the Phase 1 open-label trial of RBX7455, three dosing regimens showed an aggregate 90% CDI recurrence-free rate at 8 weeks after the last received treatment with no apparent dose–response. Responders’ microbiomes significantly shifted from before to after treatment, based on whole genome sequencing (Khanna et al., 2020). Since there was no clinical or microbiome difference among dosing groups, all three were pooled for MHI-A analysis. The median MHI-A for baseline participants was comparable to PUNCH CD2 and PUNCH Open-Label baseline values, and MHI-A for RBX7455 was similar to MHI-A for RBX2660 (Figure 2C; Supplementary Table S3). By 7 days after RBX7455 administration, MHI-A had shifted much higher, with a majority of responders >7.2 at 7, 30, 60 days and 6 months after treatment. Among non-responders, there were only five post-treatment samples (three at 7 days and two at 30 days), the majority of which were < 7.2. Thus, MHI-A data and outcomes for the RBX7455 trial, determined by whole-genome shotgun sequencing, are highly consistent with data from two RBX2660 trials, supporting that MHI-A is generalizable among multiple LBP trials as a measure of restoration.

### MHI-A for Additional Healthy Populations

**Figure 3:**
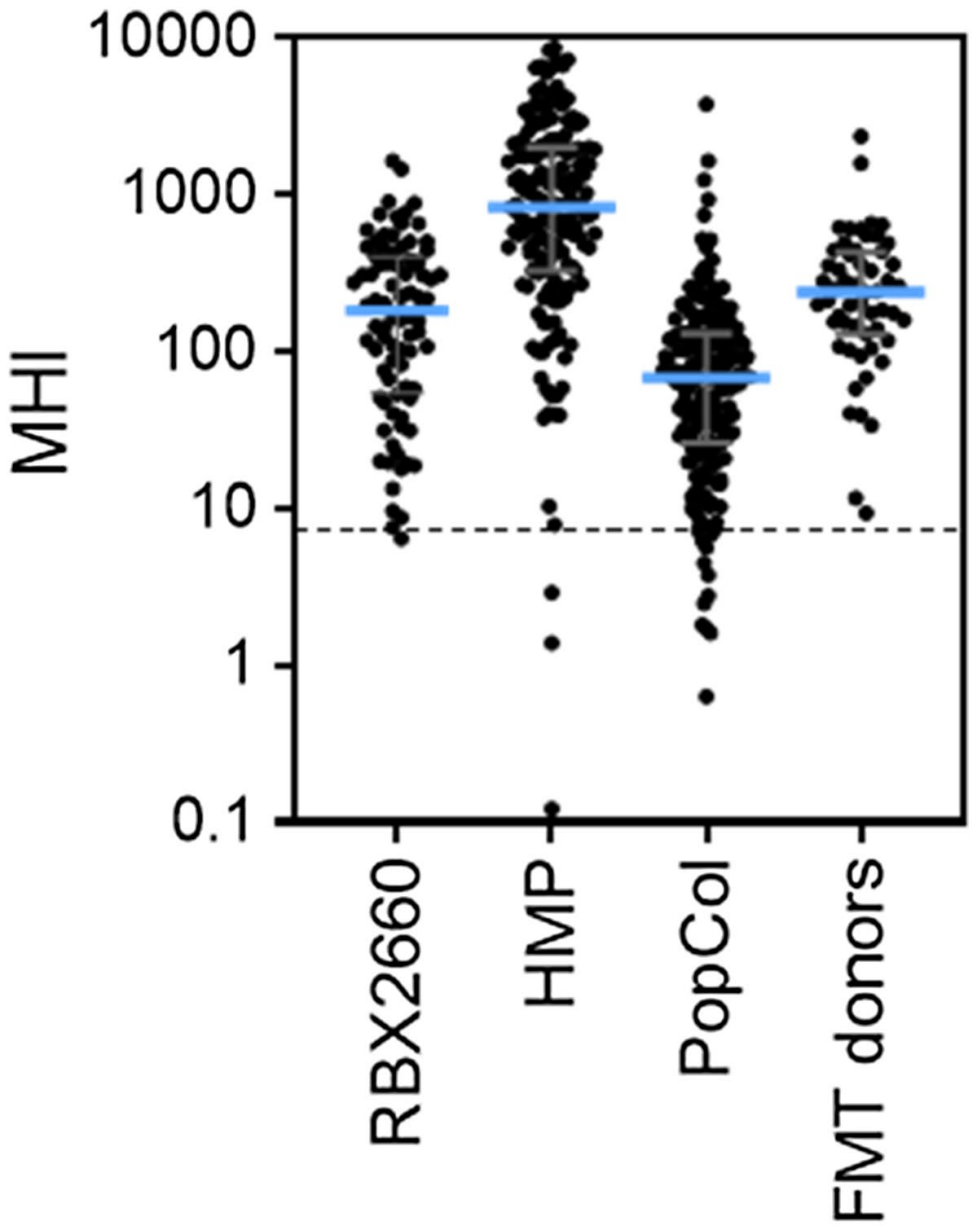
MHI-A values for RBX2660 investigational product administered in PUNCH CD2 (RBX2660) and three published healthy cohort studies, including the Human Microbiome Project (HMP), healthy Scandanavian adults (PopCol), and FMT donors from three published studies. The dotted line shows the MHI-A = 7.2 threshold, above which MHI-A values correspond to healthy based on ROC analysis.

To determine the extent to which the MHI-A range for RBX2660 is generally representative of healthy microbiota, MHI-A was calculated for several published healthy cohorts. For each, publicly deposited sequencing data was processed to MHI-A. The first comparator, the HMP, included 171 fecal samples from adults with no diagnosed systemic diseases or recent antibiotic administration, sequenced by 16S methodology. The median MHI-A for HMP was within 5-fold of the RBX2660 median, and 98% of HMP samples had MHI-A > 7.2 (Figure 3; Supplementary Table S5). The second comparator set was donor material from three independently conducted and published studies of FMT for rCDI (Weingarden et al., 2015; Khanna et al., 2017; Staley et al., 2018). The median MHI-A for these 55 samples was within 2-fold of the RBX2660 median, and 100% had MHI-A > 7.2. The third comparator set was a recently published study of non-antibiotic-treated healthy Scandinavian adults, sequenced with shallow-shotgun methods (PopCol; Hugerth et al., 2020). The median MHI-A for PopCol was within 3-fold of the RBX2660 median, and 94% of PopCol samples had MHI-A > 7.2. Collectively, these comparisons indicate that the MHI-A > 7.2 threshold is appropriately indicative of healthy microbiota populations.

### MHI-A for Antibiotic-Treated Populations

**Figure 4:**
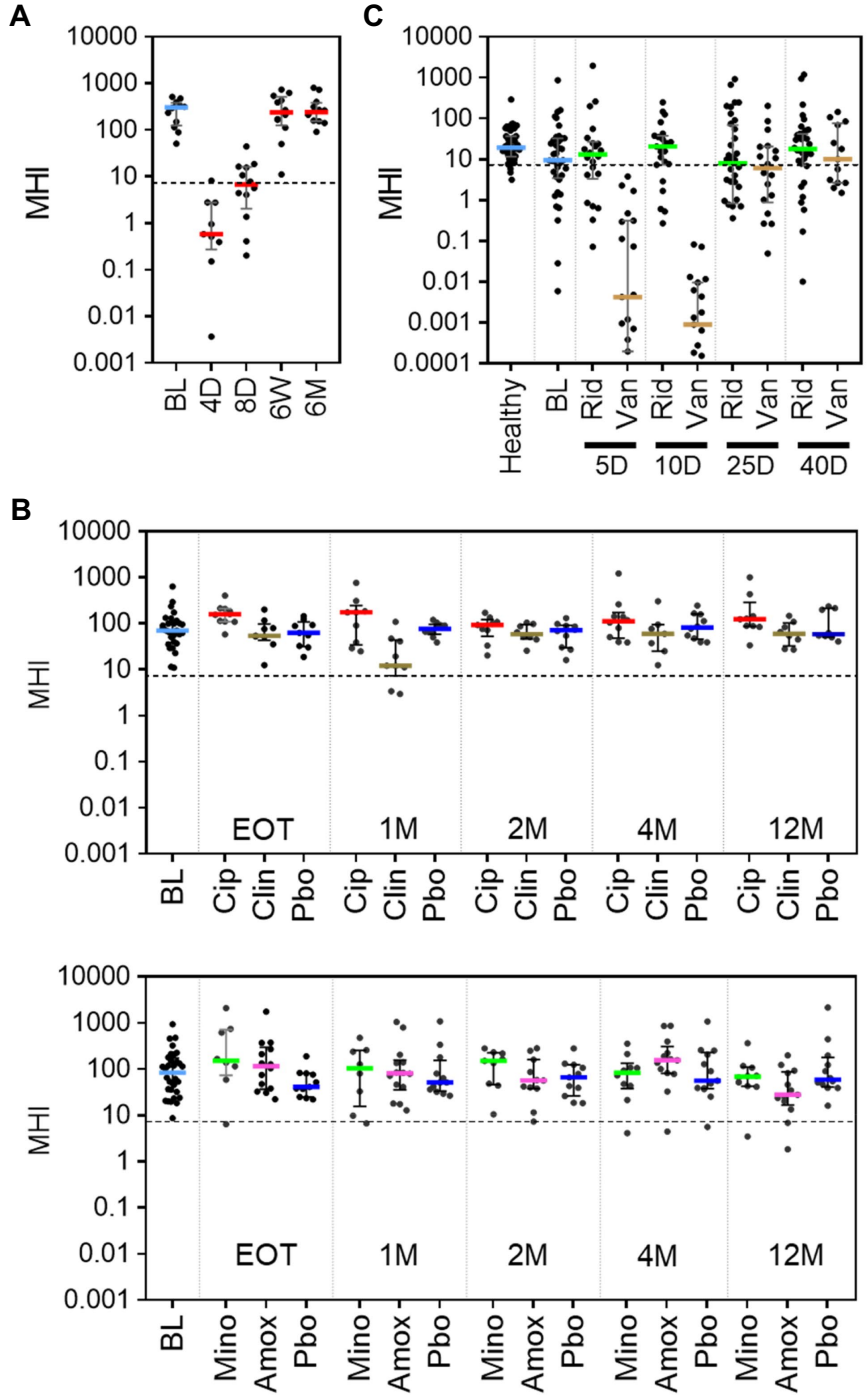
MHI-A values for patients in three published studies of the effect of antibiotics on the microbiome, shown with individual sample values, median, and interquartile range. **(A)** MHI-A values before (BL) and at 4 days (4 D), 8 days (8 D), 6 weeks (6 W), or 6 months (6 M) after the start of a 4-day course of a cocktail of three last-resort antibiotics: meropenem, gentamicin, and vancomycin. The 4-day timepoint coincided with the end of treatment. The dotted line shows the MHI-A = 7.2 threshold, above which MHI-A values correspond to healthy based on ROC analysis. **(B)** MHI-A values before (BL), at the end of treatment (EOT), and 1 month (1 M), 2 months (2 M), 4 months (4 M), or 12 months (12 M) after treatment with one of four antibiotics (ciprofloxacin, Cip; clindamycin, Clin; minocycline, Mino; or amoxicillin, Amox) or placebo (Pbo). The treatment course for all antibiotics was 1 week. **(C)** MHI-A values before (BL), and 5 days (5 D), 10 days (10 D), 25 days (25 D), and 40 days (40 D) after the start of treatment with a 10-day course of ridinilazole (Rid) or vancomycin (Van) for CDI.

To evaluate how well MHI-A can be generalized as an indicator of post-antibiotic dysbiosis, published studies of antibiotic treatments were evaluated. A literature search revealed three such studies for which longitudinal samples collected before and after antibiotic treatment were sequenced and data publicly available. For each study, sequencing data was processed to OTU from which MHI-A values were calculated. The first study characterized a 4-day course of a broad-spectrum cocktail of meropenem, gentamycin, and vancomycin in 12 healthy adult males using whole-genome sequencing (Palleja et al., 2018). Prior to antibiotic treatment, MHI-A values were > 7.2 (Pre-ABX, Figure 4A; Supplementary Table S6), but at the end of the 4-day treatment (4 D), MHI-A was much lower (*p* < 0.001) and similar to baseline participants in RBX2660 and RBX7455 trials. This MHI-A decrease was driven by lowered Bacteroidia and Clostridia abundance concurrent with a bloom of Gammaproteobacteria. By 6 weeks and 6 months after the start of treatment, MHI-A had largely recovered to pre-treatment levels. Thus, MHI-A was clearly diagnostic of a post-antibiotic microbiota shift with subsequent restoration in this study.

The second study included 66 healthy participants at two sites, who were orally administered one of four milder antibiotic treatments – ciprofloxacin, clindamycin, minocycline, or moxifloxacin (Zaura et al., 2015). The duration of treatment ranged between five and 10 days, based on treatment guidelines for each antibiotic. Samples were collected at baseline (BL), end of treatment (EOT), and 1, 2, 4, and 12 months after completion of treatment and sequenced using 16S methods. Prior to antibiotics, the median and majority of MHI-A values were > 7.2, with no significant difference between the two sites (*p* > 0.05, Figure 4B; Supplementary Table S7). Amoxicillin and minocycline did not affect MHI-A at any time point. At EOT for ciprofloxacin MHI-A was slightly higher than placebo (*p* < 0.05) but converged thereafter. The only antibiotic that significantly decreased MHI-A was clindamycin, and only at 1 month after treatment; notably, clindamycin is known to increase CDI risk (Brown et al., 2013). Overall, the milder antibiotics administered in this study caused minimal MHI-A changes.

The third study characterized the microbiome before, during, and after a 10-day course of vancomycin or ridinilazole for CDI in a Phase 2 clinical trial (NCT02092935; Thorpe et al., 2018). Ridinilazole is an investigational CDI antibiotic with a narrower spectrum and is more microbiome sparing than vancomycin, and CDI recurrence was less after ridinilazole than after vancomycin in the phase 2 trial. Before treatment, participants’ MHI-A values were slightly lower than an age-, gender-, and location-matched healthy control group and lower than RBX2660 (Figure 4C; Supplementary Table S8), as would be expected during an active CDI infection. After 5 or 10 days of vancomycin MHI-A was reduced >1,000-fold, with partial recovery by day 25 and full recovery by day 40. In contrast, ridinilazole did not significantly decrease MHI-A at any time point (*p* < 0.05). Thus, in this study MHI-A clearly differentiated between the narrower- and broader-spectrum CDI antibiotics.

## Discussion

Although clearly beneficial, antibiotics are increasingly recognized as disruptors of gut microbiota. Accordingly, biomarkers that can diagnose or monitor post-antibiotic dysbiosis and subsequent restoration are of high importance. We developed and validated the MHI-A biomarker to address this need, using three clinical trials of investigational LBPs aimed at counteracting post-antibiotic dysbiosis. In this data set, MHI-A had high diagnostic accuracy to distinguish post-antibiotic dysbiosis from healthy, and to demonstrate restoration correlated with positive clinical outcomes for reducing CDI recurrence.

The four taxonomic classes included in the MHI-A algorithm were chosen in part because they are well known to be associated with healthy versus dysbiotic communities. For instance, Bacteroidia and Clostridia help resist pathogen colonization by metabolizing primary to secondary bile salts, modulating intestinal barrier function, producing short-chain fatty acids, competing for key nutrients, engaging or activating the immune system, and other critical functions for maintaining health (Pickard et al., 2017; Ducarmon et al., 2019). Therefore, depletion of these taxa, as low MHI-A would indicate, is consistent with decreased beneficial functions. Conversely, increased abundance of Gammaproteobacteria and Bacilli, as low MHI-A would also indicate, is associated with pathologic functions like inflammation, metabolic derangement, pathogen proliferation in the gut and urinary tract, and other negative health conditions (Morgan et al., 2012; Santoru et al., 2017). Indeed, although some Gammaproteobacteria and Bacilli like *Escherichia* and *Lactobacillus* species are low-abundance commensals in healthy populations, many are critical pathogen threats whose high abundance would be self-evidently problematic, including but not limited to *Klebsiella*, *Pseudomonas*, *Enterococcus*, *Salmonella*, and *Streptococcus*. Thus, the statistically significant MHI-A difference between post-antibiotic dysbiosis and healthy reflects biologically relevant functions.

It is useful to compare MHI-A with other microbiome-based markers that have been proposed, even though none have specifically aimed to distinguish post-antibiotic dysbiosis. Alpha diversity, expressed as a Shannon index, often decreases after antibiotic treatment (Zaura et al., 2015; Thorpe et al., 2018), but the changes are usually small and can suffer from inter-sample or inter-population confounders of sequencing depth and rarefaction (Willis, 2019). Further, highly divergent compositions and/or metagenomic functions can have identical alpha diversity. The ratio of Firmicutes to Bacteroidetes phyla (F/B), often cited as a metric for predicting obesity risk, is also reported to change after antibiotics (Panda et al., 2014). Some of the MHI-A taxa, Bacteroidia, Clostridia, and Bacilli, are captured in the F/B ratio, but Gammaproteobacteria, whose increase is a hallmark of post-antibiotic dysbiosis, are not. Similarly, F/B does not distinguish beneficial from pathogenic Firmicutes. Increased beneficial Clostridia could have the same F/B as increased Enterococci, common after antibiotics or during vancomycin-resistant *Enterococcus* infections. Thus, MHI-A appears better suited than alpha diversity or F/B as a measure of post-antibiotic dysbiosis.

Another metric was proposed by Gupta and colleagues as a classifier of healthy versus general dysbiosis, based on 50 species (Gupta et al., 2020). This metric was not developed with data from antibiotic-treated patients and did not aim to identify post-antibiotic dysbiosis. Accordingly, it does not include several genera that often predominate after antibiotic treatment, like *Escherichia*, *Pseudomonas*, and *Enterococcus*. Likewise, this metric only associates one *Clostridium* species with healthy status, meaning it could not detect other Clostridia reduced by antibiotics or restored by microbiota-based treatments. Thus, while the general metric proposed by Gupta may be valuable, the MHI-A appears to contain more information related to post-antibiotic dysbiosis.

Two additional metrics were proposed to predict response to or recurrence after CDI antibiotics, and both are consistent with MHI-A. In one, elevated abundance of six taxa prior to CDI antibiotic was positively associated with successful antibiotic response (Khanna et al., 2016a). All six were Bacteroidia or Clostridia whose elevated abundance would equate to higher MHI-A. They also found 11 taxa whose elevated abundance was predictive of recurrence after antibiotic, and four of these were Bacilli or Gammaproteobacteria whose elevated abundance would equate to lowered MHI-A and lowered resistance to *C. difficile* colonization. These findings are consistent with ours and suggest MHI-A may have a role in predicting antibiotic response in CDI patients. A second study combined microbiome composition 14 days after CDI antibiotic treatment with patient clinical and demographic characteristics to predict CDI recurrence (Lee et al., 2020). They found that increased Bacteroidetes, *Lachnospiraceae*, and *Ruminococcaceae* (Clostridia), and decreased Gammaproteobacteria correlated with reduced CDI recurrence, trends that are highly consistent with MHI-A. They also cited the MHI-A described here, showing that a small change (1%) in the MHI-A reported herein was more effective at predicting CDI recurrence than changes in individual taxa alone or than the Shannon index. It may be that the ability of MHI-A to account for interconnected positively and negatively correlated taxa increases its diagnostic potential. Overall, the taxonomic changes measured by MHI-A are quite consistent with other metrics reported in the literature.

It was conceivable that taxonomic levels other than class could lead to a similar index. At the phylum level, the F/B ratio was less effective, as already described. At the order level, Clostridiales shifted concurrently with Clostridia, Lactobacillales with Bacilli, Enterobacterales with Gammaproteobacteria, and Bacteroidales with Bacteroidia. However, the accuracy of MHI-A was not increased when assessed at the order level. At the family or genus taxonomic levels, patient-to-patient variability was higher, which would decrease diagnostic accuracy. Thus, MHI-A appears simplest to calculate while retaining high accuracy.

It is intriguing to consider healthcare scenarios wherein MHI-A might be informative or diagnostically useful. Our data indicate MHI-A may rank-order antibiotics’ impact on the microbiota, which could be valuable for microbiota-sparing antimicrobial stewardship or for the development of more microbiota-sparing antibiotics. Similarly, MHI-A might be useful for demonstrating that investigational microbiota-protective therapies like ribaxamase (Kokai-Kun et al., 2019) or DAV132 (de Gunzburg et al., 2018) decrease antibiotic harm to the microbiome. MHI-A might also be useful for identifying patients at risk of dybiosis-related complications. In one cohort of hematopoietic stem cell transplant patients, *Enterococcus*, *Streptococcus*, or Gammaproteobacteria dominance of the intestinal microbiome was correlated with increased risk of bacteremia, and these microbiome signals preceded infection by up to 7 days (Taur et al., 2012). These changes would most likely be detected as reduced MHI-A. Similar correlations were observed in kidney transplant recipients (Magruder et al., 2020), for whom the risk of bacteriuria and urinary tract infections was correlated with increased Gammaproteobacteria and Bacilli and decreased Clostridia. A similar correlation is reported for liver transplant patients (Annavajhala et al., 2019). Future studies will aim to directly evaluate the utility of MHI-A in these scenarios.

MHI-A can also be considered a pharmacodynamic (PD) measure of microbiota restoration for LBPs. Typically, PD is defined as the action of a drug in the body, manifested as biochemical or physiologic effects, but debate remains about how to define PD for microbiota-based LBPs (Khoruts et al., 2021). In the trials assessed herein, MHI-A was a clear indicator compositional changes after RBX2660 or RBX7455 as well as after FMT, and these changes have biochemical and physiologic effects that have already been pointed out. MHI-A was also time- and treatment-dependent in our trials and correlated with clinical response, all hallmarks of PD markers. Accordingly, it will be useful to prospectively evaluate MHI-A as PD marker for other microbiota-restoring approaches.

A few limitations of this work bear noting. First, it is an apparent limitation that MHI-A was not decreased by all antibiotics evaluated herein, specifically in healthy volunteers that received clindamycin, ciprofloxacin, moxifloxacin, or amoxicillin. This prompts consideration of what is the minimum level of disruption detectable with MHI-A. It bears noting that other studies using culturomics or pyrosequencing methods suggested these antibiotics may be more impactful than was observed in the data set used to calculate their MHI-A shift here (Dethlefsen et al., 2008). Nevertheless, it will be important to initiate further studies to calibrate the MHI-A effects for many antibiotics, and in particular to associate which MHI-A levels correlate with specific clinical risks or outcomes. Furthermore, since our analyses were retrospective, future studies should utilize prospectively designed MHI-A endpoints or hypotheses, particularly in correlation with clinical outcomes. Third, it is unclear whether the level of MHI-A restoration observed after RBX2660 and RBX7455 is universally predictive of clinical efficacy for other investigational LBPs. It may be that less restoration could still be clinically sufficient. Evaluation of MHI-A with other investigational LBPs and correlation with clinical outcomes will be enlightening for this question.

Despite the potential limitations, there are several key strengths of our work: the large number of samples and studies assessed, the consistency of patient definitions and sample collection processes across three controlled clinical trials, the evaluation of antibiotics as well as investigational microbiota-restoring treatments, consistent performance of MHI-A among three sequencing methodologies, and the inclusion of publicly available data sets to assess the generalizability of observations. Overall, the MHI-A provides a novel perspective and promising prototype microbiome marker that merits further evaluation and development as a tool for monitoring post-antibiotic dysbiosis.

## Data Availability Statement

The datasets presented in this article are not readily available because they are restricted by an ongoing commercialization process. Requests to access the datasets should be directed to KB.

## Ethics Statement

IRB approval was obtained at each study center for NCT02299570, NCT02589847, and NCT02981316. The patients/participants provided their written informed consent to participate in this study.

## Author Contributions

KB and CJ contributed to conception and design of the study. KB, DW, CG, and WS performed the statistical analysis. KB wrote the first draft of the manuscript. All authors contributed to the article and approved the submitted version.

## Funding

The work was supported by Rebiotix, a Ferring Company, as part of a commercial clinical development program for microbiota-based investigational live biotherapeutics RBX2660 and RBX7455.

## Conflict of Interest

KB, CJ, and DW are employees of Rebiotix, A Ferring Company. CG and WS are employed by BioRankings, LLC, which received fees for this analysis from Rebiotix.

## Publisher’s Note

All claims expressed in this article are solely those of the authors and do not necessarily represent those of their affiliated organizations, or those of the publisher, the editors and the reviewers. Any product that may be evaluated in this article, or claim that may be made by its manufacturer, is not guaranteed or endorsed by the publisher.
