# Supplementary material for: Development and Validation of a Novel Microbiome-Based Biomarker of Post-antibiotic Dysbiosis and Subsequent Restoration
Source: Front Microbiol. 2022 Jan 4;12:781275. doi: 10.3389/fmicb.2021.781275 (PMC8764365; doi:10.3389/fmicb.2021.781275)
Supplement: Supplementary file 1 [file Data_Sheet_1.pdf]

Development and Validation of a Novel Microbiome-Based Biomarker of Post-Antibiotic Dysbiosis and Subsequent Restoration

Supplementary Information

Authors:
Ken Blount, PhD[1*]; Courtney Jones, BS[1]; Dana Walsh, PhD[1], Carlos Gonzalez, MS[2]; William D. Shannon, PhD[2]

[1]Rebiotix Inc., a Ferring Company, Roseville, MN, USA;[2] BioRankings LLC, St. Louis, MO, USA

Included:

Figure S1	Receiver operating characteristic analysis for PUNCH CD2
Figure S2	Longitudinal MHI-A values for PUNCH CD2
Table S1	Logistic regression analysis for taxonomic classes
Table S2	Logistic regression analysis for MHI-A
Table S3	MHI-A values for RBX2660 and RBX7455 clinical trials
Table S4	Output table of ROC analysis of MHI-A from PUNCH CD2
Table S5	MHI-A values for healthy cohorts
Table S6	MHI-A values for vancomycin-meropenem-gentamycin cocktail
Table S7	MHI-A values for ciprofloxacin, clindamycin, amoxicillin, moxifloxacin
Table S8	MHI-A values for ridinilazole and vancomycin

Figure S1. Receiver operating characteristic (ROC) analysis of MHI-A values for PUNCH CD2 participants at baseline compared to administered doses of RBX2660 drug product.

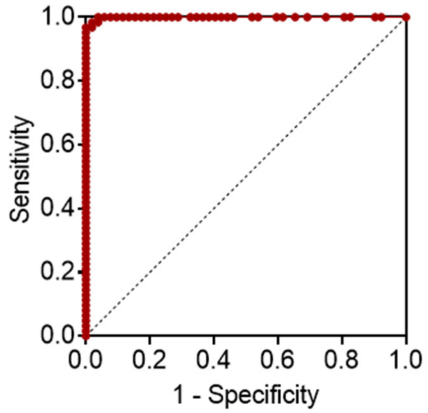

Figure S2. Longitudinal within-participant MHI-A values for the subset of RBX2660-treated PUNCH CD2 responders from whom all four displayed timepoints were received.

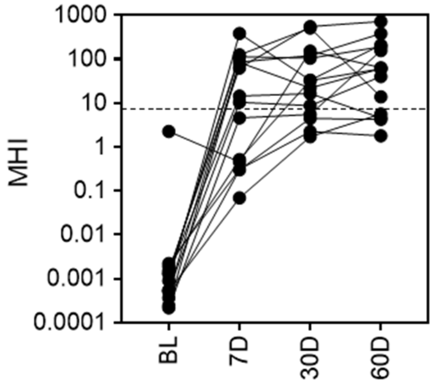

Table S1. Univariate logistic regression analysis of odds for predicting dysbiosis as a function of taxonomic classes.

Class	Coefficient (b)	Nominal P Value	Odds for Predicting Dysbiosis (exp(b))
**Univariate Tests on Individual Classes**			
Gammaproteobacteria	34.46	7.1e-5	9.2e14
Bacilli	62.40	6.5e-6	1.3e27
Bacteroidia	-25.59	1.7e-5	7.7e-12
Clostridia	-9.51	1.5e-11	7.4e-5
**Univariate Tests on Pooled Classes**			
Gammaproteobacteria + Bacilli	57.85	0.0038	1.3e25
Bacteroidia + Clostridia	-21.21	0.0003	6.1e-10

Table S2. Multivariate logistic regression analysis of odds for predicting dysbiosis as function of MHI-A ratio.

**Coefficient**	**Coefficient (b)**	**Nominal P Value**	**Odds for Predicting Dysbiosis (exp(b))**
Intercept	-6.27	0.0013	
[Gammaproteobacteria + Bacilli] / [Bacteroidia + Clostridia]	41.31	0.0093	8.7e17

Table S3. Summary of MHI-A values from RBX2660 and RBX7455 controlled clinical trial samples included in this analysis.

	Sample group	# Samples	Median MHI-A	Fraction of Samples with MHI-A >7.2
**PUNCH CD2**				
	BL	52	0.001	0.02
	RBX2660	84	183	0.99
RBX2660 Responders	7 D	29	10	0.59
	30 D	23	22	0.69
	60 D	22	96	0.77
Placebo Responders	7 D	9	1.7	0.22
	30 D	9	0.45	0.33
	60 D	10	2.7	0.30
Non-responders	7 D	5	1.7	0.20
	30 D	2	72	0.50
**PUNCH Open Label**				
	BL	111	0.006	0.05
	RBX2660	155	243	1.0
Responders	7 D	74	52	0.66
	30 D	67	23	0.71
	60 D	75	86	0.77
	6 M	63	43	0.82
	12 M	54	115	0.87
	24 M	41	94	0.83
Non-responders	7 D	10	13	0.60
	30 D	2	36	0.50
**RBX7455**				
	Baseline	24	0.01	0
	RBX7455	11	116	1.0
Responders	7 D	23	5	0.48
	30 D	25	34	0.76
	60 D	25	30	0.76
	6 M	18	51	0.78
Non-responders	7 D	3	0.8	0.33
	30D	2	4	0

Table S4. Output table of receiver operating characteristic (ROC) analysis of baseline and RBX2660 MHI values from PUNCH CD2. The shaded row highlights the cut point of 7.2, with maximum sensitivity and specificity.

Cut point	Sensitivity	Specificity	Likelihood ratio
> 5.0e-005	1.0	0.077	1.1
> 0.00015	1.0	0.096	1.1
> 0.00025	1.0	0.17	1.2
> 0.00035	1.0	0.19	1.2
> 0.00045	1.0	0.25	1.3
> 0.00055	1.0	0.31	1.4
> 0.00065	1.0	0.35	1.5
> 0.00080	1.0	0.38	1.6
> 0.0010	1.0	0.40	1.7
> 0.0012	1.0	0.46	1.9
> 0.0013	1.0	0.48	1.9
> 0.0014	1.0	0.54	2.2
> 0.0015	1.0	0.56	2.3
> 0.0017	1.0	0.58	2.4
> 0.0019	1.0	0.60	2.5
> 0.0022	1.0	0.62	2.6
> 0.0027	1.0	0.63	2.7
> 0.0032	1.0	0.65	2.9
> 0.0045	1.0	0.67	3.1
> 0.0090	1.0	0.71	3.5
> 0.016	1.0	0.73	3.7
> 0.029	1.0	0.75	4.0
> 0.048	1.0	0.77	4.3
> 0.079	1.0	0.79	4.7
> 0.11	1.0	0.81	5.2
> 0.13	1.0	0.83	5.8
> 0.47	1.0	0.85	6.5
> 1.3	1.0	0.87	7.4
> 2.0	1.0	0.88	8.7
> 2.7	1.0	0.90	10
> 3.5	1.0	0.92	13
> 3.8	1.0	0.94	17
> 5.2	1.0	0.96	26
> 6.6	0.98	0.96	26
> 7.2	0.98	0.98	51
> 9.5	0.97	0.98	50
> 12	0.97	1.0	
> 16	0.95	1.0	
> 27	0.94	1.0	
> 37	0.92	1.0	
> 45	0.90	1.0	
> 51	0.89	1.0	
> 52	0.87	1.0	
> 55	0.85	1.0	
> 58	0.84	1.0	
> 63	0.82	1.0	
> 71	0.81	1.0	
> 79	0.79	1.0	
> 86	0.77	1.0	
> 95	0.76	1.0	

> 102	0.74	1.0
> 103	0.73	1.0
> 107	0.71	1.0
> 114	0.69	1.0
> 123	0.68	1.0
> 135	0.66	1.0
> 143	0.65	1.0
> 151	0.63	1.0
> 158	0.61	1.0
> 161	0.60	1.0
> 165	0.58	1.0
> 183	0.56	1.0
> 201	0.55	1.0
> 208	0.53	1.0
> 215	0.52	1.0
> 220	0.50	1.0
> 222	0.48	1.0
> 231	0.47	1.0
> 251	0.45	1.0
> 270	0.44	1.0
> 291	0.42	1.0
> 308	0.40	1.0
> 310	0.39	1.0
> 311	0.37	1.0
> 334	0.35	1.0
> 356	0.34	1.0
> 357	0.32	1.0
> 361	0.31	1.0
> 374	0.29	1.0
> 394	0.27	1.0
> 413	0.26	1.0
> 433	0.24	1.0
> 448	0.23	1.0
> 458	0.21	1.0
> 482	0.19	1.0
> 502	0.18	1.0
> 502	0.16	1.0
> 527	0.15	1.0
> 553	0.13	1.0
> 575	0.11	1.0
> 624	0.097	1.0
> 689	0.081	1.0
> 740	0.065	1.0
> 820	0.048	1.0
> 894	0.032	1.0
> 1271	0.016	1.0

Table S5. Summary of MHI-A values from healthy studies included in this analysis.

Sample group	# Samples	Median MHI-A	Fraction of Samples with MHI-A >7.2
HMP	171	831	0.98
FMT donors	55	240	1.0
PopCol	185	68	0.94

Table S6. Summary of MHI-A values from antibiotic study of vancomycin, meropenem, gentamycin.

Sample group	# Samples	Median MHI-A	Fraction of Samples with MHI-A >7.2
BL	12	303	1.0
4 D	9	0.578	0.11
8 D[1]	12	6.72	0.5
6 W	12	238	1.0
6 M	12	240	1.0

Table S7. Summary of MHI-A values from antibiotic study of ciprofloxacin, clindamycin, amoxicillin, moxifloxacin.

	Sample group	# Samples	Median MHI-A	Fraction of Samples with MHI-A >7.2
**Site 1, Sweden**				
	BL	29	70	1.0
Cipro	1 W	10	159	1.0
	1 M	9	176	1.0
	2 M	9	93	1.0
	4 M	10	112	1.0
	12 M	9	125	1.0
Clindamycin	1 W	9	54	1.0
	1 M	9	12	0.78
	2 M	8	59	1.0
	4 M	7	60	1.0
	12 M	8	60	1.0
Placebo	1 W	9	63	1.0
	1 M	10	77	1.0
	2 M	10	71	1.0
	4 M	10	82	1.0
	12 M	8	59	1.0
**Site 2, UK**				
	BL	32	84	1.0
Amoxicillin	1 W	13	155	0.92
	1 M	13	127	1.0
	2 M	13	80	1.0
	4 M	11	57	1.0
	12 M	13	28	0.85
Minocycline	1 W	8	152	0.88
	1 M	8	104	0.88
	2 M	9	151	1.0
	4 M	10	83	0.9
	12 M	10	68	0.9
Placebo	1 W	11	41	1.0
	1 M	12	52	1.0
	2 M	11	66	1.0
	4 M	13	56	0.92
	12M	12	59	1.0

Table S8. Summary of MHI-A values from antibiotic treatment study of ridinilazole and vancomycin.

	Sample group	# Samples	Median MHI-A	Fraction of Samples with MHI-A >7.2
	Healthy1	50	19.3	0.92
	BL	37	9.4	0.62
Ridinilazole	5 D	22	13.0	0.64
	10 D	23	20.5	0.78
	25 D	36	8.0	0.50
	40 D	36	17.8	0.75
Vancomycin	5 D	19	0.004	0
	10 D	19	0.0009	0
	25 D	18	6.1	0.50
	40 D	13	10.0	0.54
